# Supplementary material for: Exercise-Induced Oxidative Stress, Nitric Oxide and Plasma Amino Acid Profile in Recreational Runners with Vegetarian and Non-Vegetarian Dietary Patterns
Source: Nutrients. 2019 Aug 13;11(8):1875. doi: 10.3390/nu11081875 (PMC6722805; doi:10.3390/nu11081875)
Supplement: Supplementary file 1 [file nutrients-11-01875-s001.pdf]

nutrients

Article – Supplementary Material

Exercise-induced Oxidative Stress, Nitric Oxide and Plasma Amino Acid Profile in Recreational Runners with Vegetarian and Non-Vegetarian Dietary Patterns

Josefine Nebl[1], Kathrin Drabert[2], Sven Haufe[3], Julian Eigendorf[3], Paulina Wasserfurth[1], Uwe Tegtbur[3], Andreas Hahn[1*], and Dimitrios Tsikas[2*]

[1] Institute of Food Science and Human Nutrition, Leibniz University Hannover, 30159 Hannover, Germany; nebl@nutrition.uni-hannover.de (JN); wasserfurth@nutrition.uni-hannover.de (PW); hahn@nutrition.uni-hannover.de (AH)

[2] Institute of Toxicology, Hannover Medical School Hannover, 30625 Hannover, Germany; Drabert.Kathrin@mh-hannover.de (KD); Tsikas.Dimitrios@mh-hannover.de (DT)

[3] Institute of Sports Medicine, Hannover Medical School, 30625 Hannover, Germany; Haufe.Sven@mh-hannover.de (SH); Eigendorf.Julian@mh-hannover.de (JE); Tegtbur.Uwe@mh-hannover.de (UT)

* Authors contributed equally to this work

Correspondence: Dimitrios.Tsikas@mh-hannover.de; Tel.: +49-511-532-3984

nutrients

MDPI

Figure S1 Flow chart of the previous study [32] from which the collected plasma samples were analyzed in the present study for MDA, nitrate, nitrite, creatinine, and the amino acids (AA).

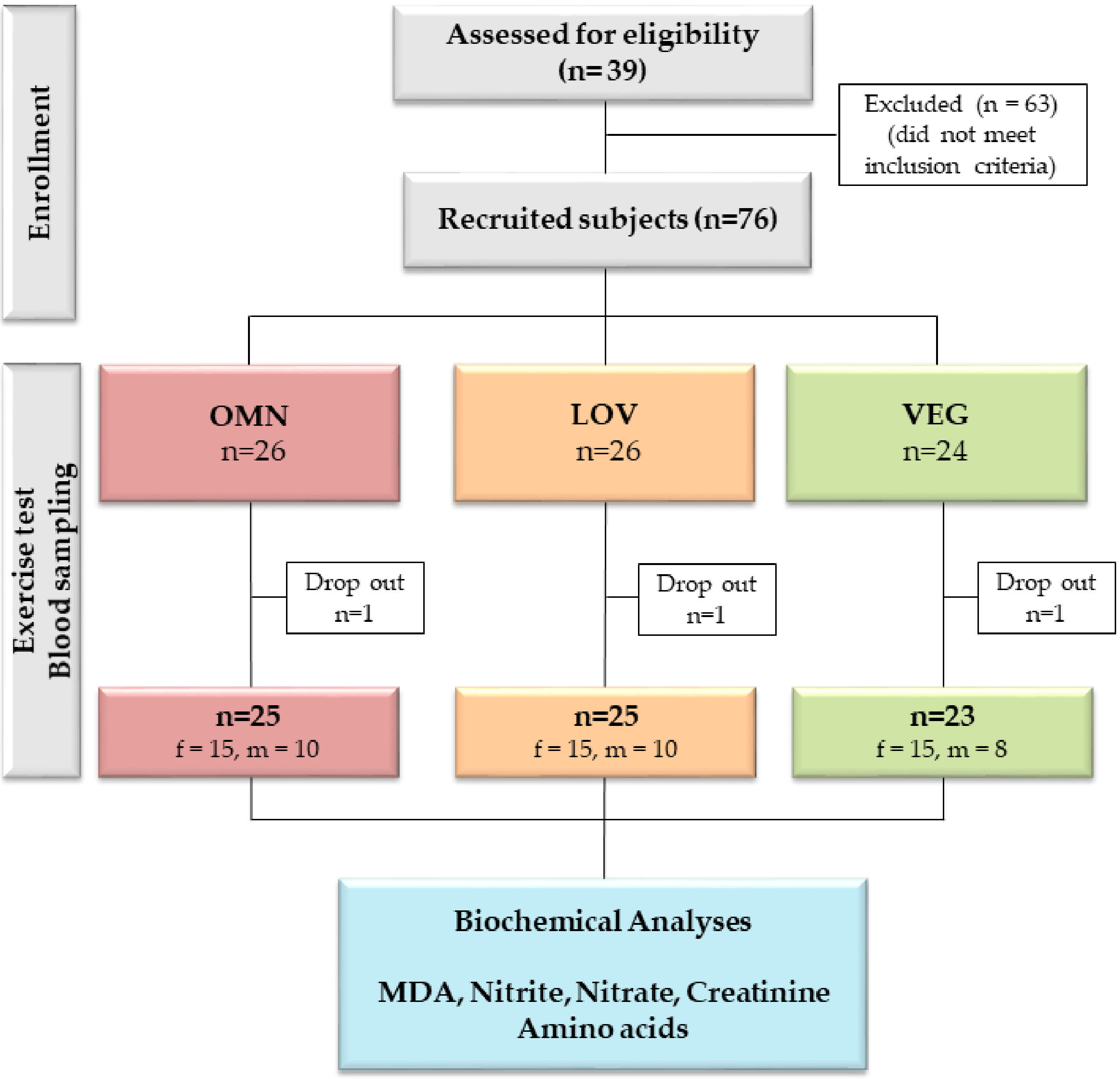

Table S1 Spearman correlation coefficients (*r*) and *p* values of the biochemical parameters pre- and post- exercise

**Parameters**	*r*	*p*
**Oxidative stress/ NO metabolism**		
MDA	0.360	**0.002**
Nitrate	0.708	**< 0.001**
Nitrite	0.444	**< 0.001**
**Kidney function**		
Creatinine	0.167	0.157
**Amino acids**		
Ala	0.802	**< 0.001**
Thr	0.797	**< 0.001**
Gly	0.861	**< 0.001**
Val	0.889	**< 0.001**
Ser	0.202	0.116
Sar	0.813	**< 0.001**
Leu+Ile	0.785	**< 0.001**
GAA	0.528	**< 0.001**
Asp+Asn	0.750	**< 0.001**
Pro	0.912	**< 0.001**
Met	0.682	**< 0.001**
Glu+Gln	0.732	**< 0.001**
Orn+Cit	0.818	**< 0.001**
Phe	0.811	**< 0.001**
Tyr	0.881	**< 0.001**
Lys	0.865	**< 0.001**
Arg	0.756	**< 0.001**
hArg	0.835	**< 0.001**
Trp	0.657	**< 0.001**
GABR	0.708	**< 0.001**
